# Open questions in functional molecular topology

**DOI:** 10.1038/s42004-020-00433-7

**Published:** 2020-12-04

**Authors:** Fredrik Schaufelberger

**Affiliations:** Department of Chemistry, University of Manchester, Oxford Road, Manchester, M13 9PL UK

**Keywords:** Interlocked molecules, Self-assembly, Supramolecular polymers

## Abstract

Molecular knots are evolving from academic curiosities to a practically useful class of mechanically interlocked molecules, capable of performing unique tasks at the nanoscale. In this comment, the author discusses the properties of molecular knots, and highlights future challenges for chemical topology.

Everyone is familiar with macroscopic knots, be they bow knots in shoelaces or stopper knots used to secure sewing threads. Since it is well known that specific knots can be tied for a desired function, it is perhaps surprising it took chemists so long to realize that knotting leads to functional advantages at the molecular level as well.

Mathematicians define all closed loops as knots, and characterize them by the number of persistent crossing points in their reduced 2D projection. In mathematical terms, a common non-interlocked macrocycle is an unknot (0_1_ in Alexander–Briggs notation, also called a topologically trivial knot), while the simplest prime knot is the three-crossing trefoil knot (3_1_)^1^. Molecular topology concerns molecules with non-planar graphs, i. e. structures that remains entangled even when the physical limitations of molecules such as bond lengths, strain and sterics are ignored. When considering the three main classes of mechanically interlocked molecules, catenanes and knots are thus topologically non-trivial, while rotaxanes are trivial (because hypothetically, stretching a macrocycle wide enough will always enable slippage)^2^.

Since the seminal synthesis of a metal-templated molecular trefoil knot by Sauvage in 1989^3^, the field has evolved immensely. In 2008, Fenlon outlined in a review what he saw as remaining open problems in chemical topology^4^. This article has served as a guideline for supramolecular chemists in the field ever since. His outlined problems were primarily concerned with synthesis: “Can we make higher-order prime knots?”, “Can a polyethylene knot be made?”, “Can a molecular Whitehead link be prepared?” Such practical considerations reflect how chemists for the past decades have focused on synthetic problems rather than questions of functions and applications of molecular topology. As it previously was so difficult to make knots at the nanoscale, it was also natural to concentrate research on synthetic aspects. However, in recent years many new topologies such as 4_1_^5^, 5_1_^6^, 5_2_^7^, 7_1_^8^, 8_18_^9^, and 8_19_^10^ prime knots have finally been constructed (Fig. 1). Knot synthesis is now becoming more routine, much like for rotaxanes and catenanes at the turn of the last century. Additionally, many of Fenlon’s questions have been answered and a wealth of methods for accessing novel knot scaffolds have been developed. The time is thus ripe for molecular topologists to ask why molecular knots are interesting and what we can use them for. This text provides a brief overview of current progress and future directions in the field.

**Fig. 1:**
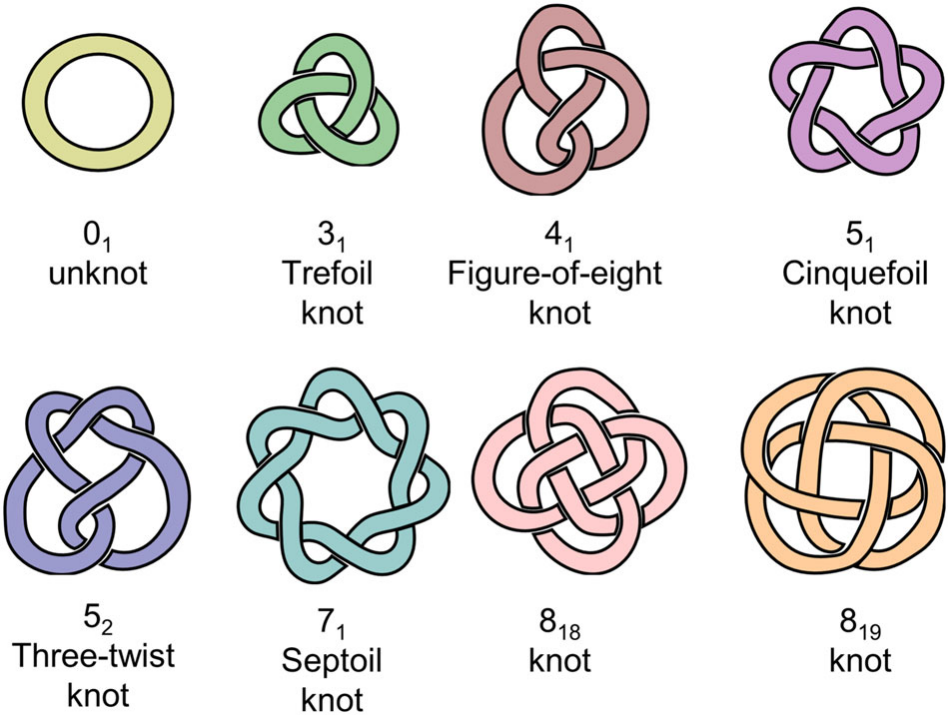
Prime knot topologies synthesized at the molecular scale to date. The knots shown here are reduced 2D projections of the corresponding molecular structures.

## What useful functions do synthetic molecular knots have?

**Fig. 2:**
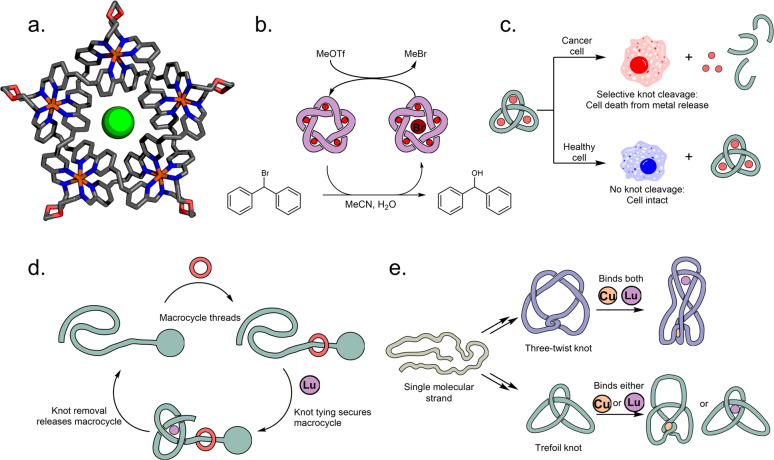
Functions of molecular knots. **a** X-ray crystal structure of a molecular pentafoil knot that can bind halide ions strongly within its central cavity. **b** Molecular knots performing catalysis, here illustrated by a pentafoil knot binding bromide and catalysing a hydrolysis reaction (MeOTf is converted to gaseous MeBr to complete the catalytic cycle and enable turnover). **c** Trefoil knots act as delivery agents of metals (Cu^II^, Fe^II^, Mn^II^, Zn^II^, Cd^II^) that selectively kill cancer cells. **d** A molecular knot can be used to perform mechanical functions at the nanoscale, such as securing and releasing a macrocycle. **e** Several different knots can be tied from the same molecular strand. The resulting topoisomeric three-twist and trefoil knots possess different metal binding properties.

Until recently, the study of functional molecular knots was essentially undeveloped. Leigh and co-workers demonstrated the first practical function of an entangled molecular strand by showing that an imine pentafoil 5_1_ knot acts as an extremely strong anion binder, with chloride binding affinities approaching those of Ag^I^ (Fig. 2a)^6^. This led to the application of a pentafoil knot as an anion-binding catalyst, with orders of magnitude rate accelerations for hydrolysis, Diels-Alder and Michael reactions (Fig. 2b)^11^. Recently, Trabolsi and co-workers demonstrated the first use of synthetic knots in chemical biology by using an imine-based trefoil knot as a delivery vector for metal ions that induce apoptosis upon release in the cellular environment (Fig. 2c)^12^. The knots showed good selectivity for human cancer cells over noncancer cells in vitro, and were significantly more potent than the well-established chemotherapeutic agent cisplatin.

Early knot syntheses relied on complex multicomponent assembly, which usually only enabled access to highly symmetrical structures. If instead binding patterns are programmed into a preconstructed backbone (much like a protein peptide or DNA nucleobase sequences), it is possible to use simple stimuli to fold a single strand around itself into a knotted shape^13^. In particular, folding of tris(2,6-pyridinedicarboxamide) ligands around lanthanide ion templates has proven to be highly useful thanks to its dynamic nature, meaning the knotting event can be used as a molecular switch^14^. Alternating between entangled and loose states is possible by treating the system with lanthanide ions and hard bases, respectively. This switching system was used in the first example of interfacing knots with molecular machines. By using the bulk generated upon knotting, it was possible to stopper a pseudo-rotaxane architecture reversibly and kinetically trap a macrocycle on a thread (Fig. 2d)^15^.

Tying a knot into a strand also introduces elements of helical and topological chirality. Recently, the Leigh and Katsonis groups employed the change in chiral expression between an enantiopure point chiral strand and its corresponding enantiopure knot to switch helical screw sense in cholesteric liquid crystals^16^. This nanoscale knotting event was used to reversibly invert the chiral organisation of the material up to the centimetre scale—an amplification event over seven orders of magnitude. These examples show that the dynamic behaviour of molecular knots can be practically useful. Given the importance of controlled molecular movement in other mechanically interlocked architectures such as rotaxanes^2^, it is reasonable to suggest molecular knots will find many future applications.

### What are the biological functions of knots?

An often-cited justification for studying molecular knots is the ubiquitous presence of entanglements in nature, both at the macro (animals using knotting to secure nests), meso (knotted hyperstructures in chromatin strands) and molecular (knotted DNA and proteins) scales^17^. It has been clear for two decades that knots are prevalent across a wide range of DNA hyperstructures as well as in many protein families. Yet, it would appear that nature actively tries to rid itself of such entanglements. For example, topoisomerase enzymes have evolved with the function to untie knots which have accidentally formed during supercoiling of DNA. Only 1–2% of proteins have topologically non-trivial tertiary structures, which is far less than predicted for such long and flexible chains based on statistical entangling^18^. So what are molecular knots really used for in nature? Knotting reduces degrees of freedom of a molecular strand and it is easy to speculate that this is useful for stabilizing catalytically active conformations in enzymes. Similarly, it has been suggested that the bulk gained by knotting protects entangled proteins from degradation by preventing uptake into the proteasome through nanosized pores^19^. However, there are no definitive answers to what—if any—functions knotting actually have in nature or if knotted structures are “just another conformation” that long chains can adopt.

### Is a certain knot topology best suited to a specific function?

At the macroscale it is well-recognized that different knots are suited for different functions—you would not try to secure a boat to a mooring with the same knot used to tie a decorative ribbon! It has long been speculated that similar functional differences between knots exists at the molecular level, and to link particular topologies to specific functions is a major goal of chemical topology. However, this has been hindered by the inability to synthesize topoisomeric knots, i.e. macrocycles with identical backbones but different three-dimensional topologies. The first system where a single molecular strand could be selectively tied into three different knots—an unknot, trefoil knot and three-twist 5_2_ knot—was recently disclosed (Fig. 2e)^7^. Divergent knotting was achieved by introducing alternating and orthogonal coordination sites for a transition metal (Cu^I^) and lanthanide (Lu^III^). It was then demonstrated for the first time that knot topologies can have different functions—while the tightly knotted 5_2_ knot can simultaneously accommodate both a transition metal ion and a lanthanide ion, the looser 3_1_ knot is topologically restricted and can only accommodate one at a time (and as a result of the metal binding the knot conformation changes). Changing the topology of a strand might be useful for manipulating its mechanical strength or changing its binding affinity towards a specific guest, but further research in this area is necessary.

### How does knotting change the mechanical properties of a strand?

Spontaneous entanglements occur on agitation of any flexible chain beyond a certain length, such as when putting earphones into a pocket. This extends to the molecular level, where Brownian motion drives the formation of knots in DNA and synthetic polymers^1^. However, almost nothing is known about what happens when molecular knots are put under applied force. Theoretical studies indicate that bonds in molecular knots are weakened at the apex of the entanglement due to distortion of covalent bonds^20^, but this has not been confirmed experimentally. Would knotted materials also be mechanically weakened? In such a case, can that strain be practically useful, for example for activating specific bonds towards cleavage? These questions also have practical implications: for the promising technology of nanopore DNA sequencing, pore clogging by DNA knots has long been implicated as a potential problem^21^.

### What are the effects of knot tightness?

Aside from the identity of the knot being tied (i.e. number of crossing points and their connectivity), tightness is the key parameter that determines the properties of a knotted strand. Recently, a systematic study of 8_19_ knots with varying degrees of tightness provided empirical evidence of this^22^. Aside from intuitive results, for example that tighter knots occupy smaller conformational space, it was also found that more tightly wound knots display significantly stronger chiral expression and fragment more easily in collision-induced dissociation mass spectrometry. Cougnon also found that more-entangled knots and links have stronger chiral expression compared to their less-entangled counterparts^23^ and Schalley noticed that knots have far less conformational freedom compared to unknotted macrocycles when measured with ion mobility collision-induced dissociation mass spectrometry^24^. Several key questions in this area remain to be answered, however. At which tightness levels do typical properties arising from entangling start to appear? What is the tightest knot that can be tied? Can stochastic knotting be used for rationally designed entanglements (i.e. kinetically controlled knotting)? Can the thermodynamics of knotting be better understood?

### How do conformational dynamics affect knots?

Many complex topologies are formed through constitutional dynamic self-assembly, for example via metal–ligand coordination interactions^8,9,25^ or dynamic covalent bonds^5,6,12^. However, dynamics are also important for knots beyond purely synthetic aspects. A range of effects stem from conformational restriction due to knotting. For example, reptation is the snake-like motion of chains^26^, a phenomenon that generally gives rise to spectral broadening and averaging, as for example observed in the spectacular all-benzene trefoil knot from Itami^27^. However, the precise consequences of reptation in knots remain to be studied. Some knots (such as 4_1_ and 8_18_ entanglements) are amphichiral, i.e. they have chiral representations that can be deformed continuously into each other, so have an overall achiral topology. This effect is underexplored, and it is of high interest to understand racemization barriers between the representations and possibly prepare persistent (trapped) chiral conformations of amphichiral knots.

## Outlook

The unique changes that result in a strand upon knotting—the increase in local bulk and introduction of topological chirality, for example—are elements that surely will be useful for constructing functional dynamic systems in supramolecular chemistry. To further explore knot functions and expand the field, there is still a need to simplify synthesis and increase the accessibility of knotted architectures. However, entry to molecular knots has now improved to the point where properties of this intriguing class of molecules can finally be studied in more detail. Rapid progress in this area is anticipated over the next few years, as scientists discover how knots can be as useful on the nanoscale as in the macroscopic world around us.
